# Interaction Mechanism of Flavonoids and α-Glucosidase: Experimental and Molecular Modelling Studies

**DOI:** 10.3390/foods8090355

**Published:** 2019-08-21

**Authors:** Chengyun He, Xiaoling Liu, Zhaojing Jiang, Sheng Geng, Hanjun Ma, Benguo Liu

**Affiliations:** 1 School of Food Science, Henan Institute of Science and Technology, Xinxiang 453003, China; 2 Key Laboratory of Biorheological Science and Technology, Ministry of Education, School of Bioengineering, Chongqing University, Chongqing 400044, China

**Keywords:** flavonoids, α-glucosidase, interaction, molecular docking, independent gradient model

## Abstract

Flavonoids are known to play a role in hypoglycemia by inhibiting α-glucosidase. However, their interaction mechanism with α-glucosidase still needs to be elaborated. In this study, the α-glucosidase inhibitory activities of 15 flavonoids were investigated. Their molecular volume had a negative effect on inhibitory activity, while the number of phenolic hydroxyl groups on the B ring was positively correlated with inhibitory activity. To explain the significant differences in activity, the interaction behaviors of myricetin and dihydromyricetin, which have similar structures, were compared by spectrofluorimetry, molecular docking, and the independent gradient model (IGM). In the fluorescence analysis, myricetin exhibited a higher binding capacity. Based on molecular docking and IGM analysis, their non-covalent interactions with α-glucosidase could be visualized and quantified. It was found that they had different binding modes with the enzymes and that myricetin possessed stronger hydrogen bonding and van der Waals force interactions, which explained the thermodynamic results.

## 1. Introduction

Diabetes mellitus is one of the most serious and chronic diseases characterized by hyperglycemia, which can lead to many complications including nephropathy, neuropathy, and cardiovascular diseases [1,2]. Today, its incidence is rising rapidly with the ageing population and increase in obesity. Its incidence is predicted to increase from 2.8% (171 million) in the year 2000 to 4.4% (366 million) by the year 2030 [3].

While diabetes places a burden on patients, its management and treatment costs have also become a huge burden on human society. It has been reported that type II diabetes accounts for more than 90% of diabetes cases, and postprandial blood glucose plays a key role in the development of type II diabetes and its complications [4]. One effective strategy for controlling postprandial hyperglycemia is to delay glucose absorption by using α-glucosidase inhibitors [5]. Synthetic α-glucosidase inhibitors (acarbose, miglitol, and voglibose) all have drawbacks such as hepatotoxicity and adverse gastrointestinal symptoms, which necessitate the search for safer and more effective alternatives [3].

In the past 20 years, plant-derived flavonoids have attracted extensive attention due to their various biological activities, including their antioxidant, antiviral, anti-inflammatory, anti-tumor, and anti-cardiovascular activities [6,7,8]. Flavonoids can regulate glucose absorption and homeostasis, with disaccharidases as targets. It has been reported that after two days of treatment with myricetin (3 mg/12 h), hyperglycemia in diabetic rats was reduced by 50% [9]. Dihydromyricetin can improve skeletal muscle insulin resistance by inducing autophagy via the AMPK signaling pathway [10]. Many flavonoids, such as quercetin and kaempferol, have exhibited α-glucosidase inhibitory activities [11,12]. Proenca et al. (2017) found that flavonoids with two catechol groups at the A or B ring and a hydroxy group at C3 possessed the highest α-glucosidase inhibitory activity [13]. The absence of a double bond between C2 and C3 and a ketonic group at C4 significantly reduces the α-glucosidase inhibitory activity of flavonoids [14].

Flavonoids exert their α-glucosidase inhibitory activities by forming complexes with enzymes through non-covalent interactions [15]. During this process, the enzyme molecules undergo structural changes and energy transfer, which can be detected by common experimental methods (fluorescent spectrometry, circular dichroism, isothermal titration calorimetry, surface plasmon resonance, etc.) [16,17,18]. However, these complicated measurement conditions introduce great difficulties into the experimental design and analysis, and most of the experimental technologies have failed to obtain a detailed structure of the complexes and to quantitatively visualize the non-covalent interactions.

In recent years, computational methods have gradually become important research tools. Molecular simulation methods such as ONIOM calculations, molecular dynamics, and molecular docking have been widely used to study the binding modes and energies of bioactive compounds and enzymes. Zeng et al. (2016) investigated the binding modes of two isoflavones (daidzein and genistein) with trypsin using molecular docking methods [19]. Li et al. (2009) clarified the interaction mechanism of the cyclin-dependent kinases (CDK2 and CDK4) and their inhibitor (2PU) based on ONIOM calculations [20]. In 2017, Lefebvre et al. presented a new approach, the independent gradient model (IGM), which enabled the automatic identification and visualization of covalent and non-covalent interactions of atoms/fragments in the 3D real space using pro-molecular density [21].

In this study, the α-glucosidase inhibitory activity of 15 flavonoids from edible plant-derived foods were compared. Then, the interaction behaviors of myricetin and dihydromyricetin with α-glucosidase were investigated by spectrofluorimetry. The underlying interaction mechanism was also clarified based on the molecular docking and IGM methods.

## 2. Materials and Methods

### 2.1. Materials and Chemicals

The 15 flavonoids (myricetin, fisetin, quercetin, baicalein, luteolin, isoliquiritigenin, chrysin, dihydromyricetin, 4-hydroxychalcone, 4′-hydroxychalcone, hesperetin, naringin, hesperidin, baicalin, rutin) and acarbose were purchased from Aladdin (Shanghai, China). α-Glucosidase from *Saccharomyces cerevisiae* and p-nitrophenyl-α-d-glucopyranoside (pNP-G) were the products of Sigma-Aldrich (St. Louis, MO, USA). The ultrapure water was obtained from a Thermo Gen Pure UV/UF water system (Waltham, MA, USA).

### 2.2. α-Glucosidase Inhibitory Assay

The α-glucosidase inhibitory activity of the flavonoids was determined according to the methods described in a previous report [22]. The substrate, pNP-G, can be hydrolyzed by α-glucosidase to p-nitrophenyl (pNP), with a maximum absorbance peak at 405 nm. For the inhibition assay, the α-glucosidase, pNP-G, and flavonoids were dissolved in a phosphate buffer (0.1 M, pH 6.8), then 1 mL of each flavonoid at different concentrations and 1 mL of 0.2 U/mL α-glucosidase solution were mixed and incubated at 37 °C for 10 min. Next, 1 mL of a 1 mM pNP-G solution was added. The obtained mixture was kept at 37 °C for 20 min. Then, 1 mL of ethanol was added to terminate the reaction. The absorbance of the mixture at 405 nm (A_sample_) was measured using a UNICO 7200 spectrophotometer (Shanghai, China). The absorbance of the mixture containing PBS instead of a flavonoid was also read as the A_control_. The α-glucosidase inhibitory activity of the flavonoids was calculated by Equation (1):

(1) $$\alpha -\mathrm{Glucosidase}\;\mathrm{inhibitory}\;\mathrm{activity}=\frac{A_{\mathrm{control}}{-A}_{\mathrm{sample}}}{A_{\mathrm{control}}}\times 100\%$$

### 2.3. Determination of Interaction Behaviors of Myricetin and Dihydromyricetin

To clarify the α-glucosidase inhibitory mechanism of flavonoids, myricetin and dihydromyricetin were selected for fluorescence measurement. They have similar structures but exhibit very different α-glucosidase inhibitory activities. The fluorescence spectra of α-glucosidase in the presence of myricetin or dihydromyricetin at different concentrations and at 25 or 30 °C were recorded by an Agilent Cary Eclipse fluorescence spectrophotometer (Santa Clara, CA, USA), with the excitation and emission slits set at 5 nm. The excitation wavelength was set at 280 nm, while the range of the emission wavelength was 300–440 nm.

### 2.4. Molecular Docking Method

The 3D structure of the α-glucosidase from *Saccharomyces cerevisiae* (PDB, 3A4A) was downloaded from the RSCB protein databank (http://www.rcsb.org). Before docking, the structure of the α-glucosidase was prepared by removing the water and ligands, adding polar hydrogen and charge, and repairing residues. The chemical structures of myricetin and dihydromyricetin were established and optimized using MOPAC 2016 and employing a semiempirical PM6 method [23]. By using Autodock 4.2 software [24], myricetin and dihydromyricetin were docked into the active site based on the binding mode of maltose to the enzyme by the Lamarckian genetic algorithm method. The grid spacing was set at 0.375 Å, and all of the points of the grid box in the x, y, and z directions were 60. During docking, the rigid structure of the α-glucosidase was maintained while the structure of the flavonoid was regarded as fully flexible. Then, the complex with the lowest binding energy was selected and analyzed to obtain a schematic diagram of the interaction between the flavonoid and α-glucosidase by using LigPlot+ v.2.1 software [25].

### 2.5. IGM Method

For the IGM analysis, the PDB file containing the molecular docking result was imported into the Multiwfn 3.6(dev) software [26]. The flavonoid and α-glucosidase were defined as fragments. The whole system was also covered by a high-quality grid (approximately 1,728,000 points in total). Then, Multiwfn started to calculate the density gradient of atoms (δ_g_), the electron density, and the sign of the second Hessian eigenvalue in Laplacians sign(λ_2_)*p* in real space based on the pro-molecular density. The obtained δ_g_ could be separated into δ_ginter_ and δ_gintra_, representing the interfragment and intrafragment interactions, respectively. Based on these grid data, the non-covalent interactions between the flavonoid and α-glucosidase could be visualized and quantified using VMD software [27].

### 2.6. Statistical Analysis

The α-glucosidase inhibitory assay was carried out based on three independent experiments. Statistical comparisons were performed using the t-test. A *p* value of <0.05 was considered to be a significant difference.

## 3. Results and Discussion

### 3.1. α-Glucosidase Inhibitory Activities

As shown in Table 1, the α-glucosidase inhibitory activities of the 15 flavonoids were compared. A significant effect of the chemical structures of flavonoids on their inhibitory activity was found. The preliminary structure–activity relationship (SAR) was summarized as follows: (1) Compared with the flavonoid aglycones (Compounds 1–10), the flavonoid glycosides (Compounds 11–15) exhibited poor inhibitory activity; (2) for the flavonoid aglycones, the number of phenolic hydroxyl groups on their B ring had a positive effect on their inhibitory activity. The former might be due to the large molecular size of flavonoid glycosides, which hindered their access to the enzyme. The latter could be explained by the B ring of flavonoids pointing to the inside of the active center of the enzyme, which is an important site for their binding with the enzyme. A strange phenomenon was also observed in that, although myricetin and dihydromyricetin had similar structures, they differed in their inhibitory activity by more than 40 times, which could not be explained solely by the difference in molecular volume and the number of phenolic hydroxyl groups on their B ring. Therefore, myricetin and dihydromyricetin were selected for further study.

### 3.2. Fluorescence Analysis

**Figure 1:**
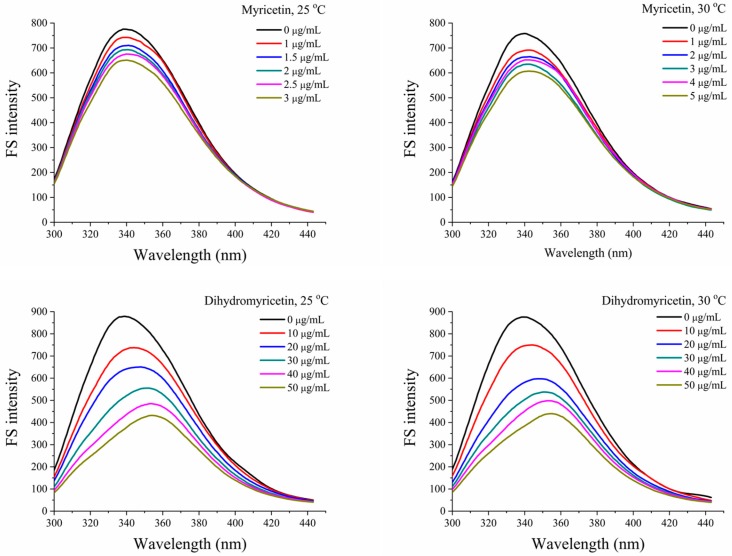
Effects of myricetin and dihydromyricetin on the fluorescence spectra of α-glucosidase at 25 and 30 °C (FS: Fluorescence Spectrum).

In this study, the interactions of myricetin and dihydromyricetin with α-glucosidase were systematically investigated by spectrofluorimetry [28]. In Figure 1, with the addition of the flavonoid, the intrinsic fluorescence of α-glucosidase at 25 and 30 °C gradually decreased. The corresponding quenching rate constant (K_q_) could be calculated based on the following Stern–Volmer equation:

F_0_/F = 1 + K_sv_[Q] = 1 + K_q_τ_0_[Q], (2)

where F_0_ and F are the peak fluorescence intensities of α-glucosidase in the absence and presence of the flavonoid; [Q] is the concentration of the flavonoid; and τ_0_ is the average life of the proteins (10^−8^ s).

In Table 2, the K_q_ values of both myricetin and dihydromyricetin were higher than the maximum K_q_ value for dynamic quenching (2 × 10^10^ L∙mol^−1^∙s^−1^), indicating that they caused fluorescence quenching by a static quenching mechanism; that is, by forming complexes with the enzyme. Then, a double-logarithm Equation (3) was applied to obtain the corresponding binding constant (K_a_) and binding site number (*n*) (Table 2):

lg[(F_0_ − F)/F] = lgK_a_ + *n*lg[Q]. (3)

For myricetin and dihydromyricetin, their binding site numbers with α-glucosidase were near 1, which suggests that they interacted with the enzyme at a molar ratio of 1:1. As the temperature increased, their binding constants decreased, because the increased temperature was able to destroy the non-covalent interaction and reduce the stability of the complex.

To determine the non-covalent interaction types, the thermodynamic parameters (enthalpy change, ΔH; entropy change, ΔS; free-energy change, ΔG) were calculated by Equations (4)–(6):

(4) $$\ln\frac{K_{a2}}{K_{a1}}=\frac{\Delta H}{R}(\frac{1}{T_{1}}-\frac{1}{T_{2}})$$

(5) $$\Delta {G=-\mathrm{RTlnK}}_{a}$$

(6) $$\Delta S=\frac{\Delta H-\Delta G}{T}$$

As shown in Table 2, both of the ΔG values were negative, indicating that the binding of myricetin and dihydromyricetin with α-glucosidase was a spontaneous process. The corresponding ΔH and ΔS values were also negative. According to a previous report [29], ΔH > 0 and ΔS > 0 meant that the hydrophobic interaction is the main force, and ΔH < 0 and ΔS < 0 implied that hydrogen bonding and van der Waals forces played the primary role, while ΔH < 0 and ΔS > 0 suggested that electrostatic forces were driving the binding. It could be concluded that the complexes of myricetin and dihydromyricetin with α-glucosidase were maintained by hydrogen bonding and van der Waals forces.

### 3.3. Molecular Docking Analysis

**Figure 2:**
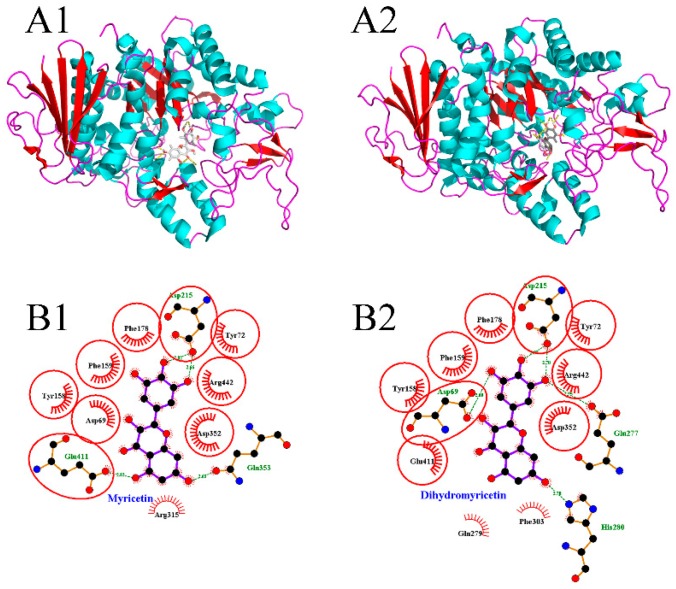
Molecular docking results of myricetin and dihydromyricetin with α-glucosidase ((**A1**,**A2**) are the binding modes of myricetin and dihydromyricetin with α-glucosidase, respectively; (**B1**,**B2**) are the schematic diagrams of the interactions between myricetin and dihydromyricetin with α-glucosidase, respectively).

The molecular docking method has been widely applied to investigate the binding modes between enzymes and inhibitors [30,31]. To clarify the significant differences between myricetin and dihydromyricetin in terms of their α-glucosidase inhibitory activity, their 3D binding modes with α-glucosidase (Figure 2A1,A2) were established by using Autodock 4.2 software. It was found that the active center of α-glucosidase was located inside the enzyme molecule. As a result, when the flavonoid glycosides with a larger molecular volume entered, the steric hindrance to be overcome was greater than that of flavonoid aglycones, so the inhibitory performance of flavonoid glycosides was generally lower. Figure 2B1,B2 shows the schematic diagrams of the interactions between flavonoids and α-glucosidase. The equivalent residues have a red underlay beneath their bonds and atoms. It was found that: (1) Asp 215 was involved in the hydrogen-bonding interaction between each flavonoid with the enzyme; and (2) Tyr 72, Tyr 158, Phe 159, Phe 178, Asp 352, and Arg 442 had hydrophobic interactions with both myricetin and dihydromyricetin. Although the structures of myricetin and dihydromyricetin only differed in the bond types between C2 and C3, their hydrogen-bonding interactions with α-glucosidase were quite different. Dihydromyricetin could form hydrogen-bonding interactions with more amino acid residues (Asp 69, Asp 215, Glu 277, His 280), but the average length of the hydrogen bonds was only 2.84 Å, which was significantly inferior to that of myricetin (2.74 Å), and it lacked hydrogen bonding in the zone around C5. The corresponding lowest binding energies for myricetin (−9.6 kcal/mol) and dihydromyricetin (−9.56 kcal/mol) were very close. Therefore, it was difficult to directly determine which flavonoid had the stronger binding capacity. Other theoretical methods are needed to quantitatively compare their interactions with α-glucosidase based on the molecular docking results.

### 3.4. IGM Analysis

**Figure 3:**
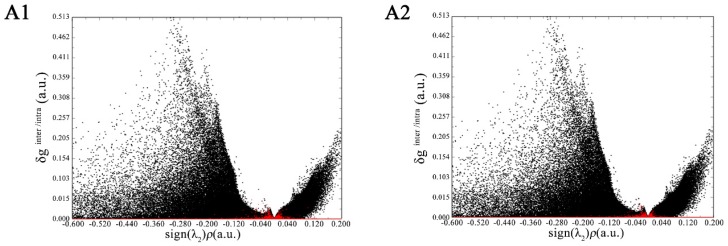
Visual scatter maps between δ_ginter_ and δ_gintra_ vs. sign(λ_2_)*p* of myricetin (**A1**) and dihydromyricetin (**A2**) with α-glucosidase.

The visualization and quantification of the interaction based on IGM is a new development in molecular simulations [21]. The δ_g_ is a key function in the IGM analysis framework; it is designed to reveal interactive regions between two (or even more) fragments. It can be separated into δ_ginter_ and δ_gintra_, which can reflect the interfragment and intrafragment interactions, respectively. Figure 3 simultaneously shows the scatter graphs of δ_ginter_ and δ_gintra_ vs. sign(λ_2_)*p*, in which the red and black points correspond to δ_ginter_ and δ_gintra_, respectively. In Figure 3A1,A2, there is a very prominent black peak of δ_gintra_ around sign(λ_2_)*p* = −0.3, representing the strong intrafragment covalent-bonding interactions. In the region where sign(λ_2_)*p* is about −0.04, δ_ginter_ has a remarkable red peak with a height of approximately 0.03, which implies the existence of hydrogen bonding [21]. In the region where sign(λ_2_)*p* is approximately +0.02, there is also a small red peak of δ_ginter_. Since a positive sign(λ_2_)*p* implies a repulsive interaction, the peak could reflect weak steric regions between the flavonoid and α-glucosidase. For myricetin, there are more red points around the sign(λ_2_)*p* of −0.04, indicating its stronger hydrogen-bonding interaction with α-glucosidase.

**Figure 4:**
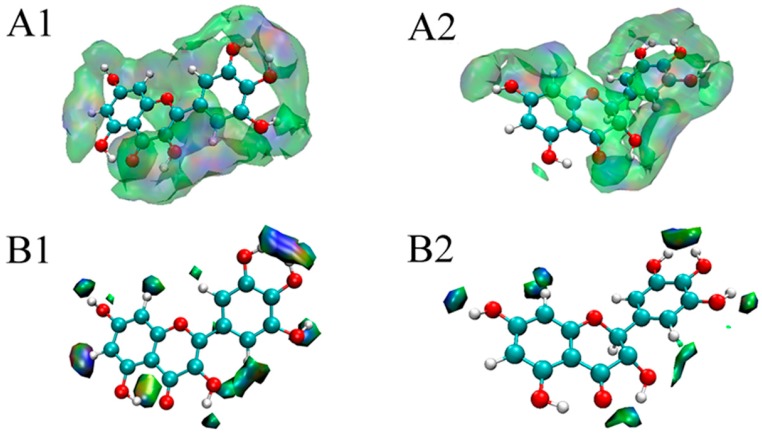
Visual weak interactions of myricetin and dihydromyricetin with α-glucosidase ((**A1**,**A2**) are the color-filled δ_ginter_ isosurfaces around myricetin and dihydromyricetin at an isovalue of 0.001, respectively; (**B1**,**B2**) are the color-filled δ_ginter_ isosurfaces around myricetin and dihydromyricetin at the isovalue of 0.01, respectively).

By exporting the grid data of sign(λ_2_)*p*, δ_g_, and δ_ginter_ to the VMD program, the color-filled δ_ginter_ isosurfaces could be drawn. Figure 4A,B show the δ_ginter_ isosurfaces at the isovalues of 0.001 and 0.01 for myricetin and dihydromyricetin, respectively. The color transition was blue–green–red. The more blue the isosurface was, the stronger the attractive interaction; however, the more red the isosurface was, the larger the steric effect. A green zone in an isosurface indicates van der Waals interactions. As shown in Figure 4A1,A2, both myricetin and dihydromyricetin were surrounded by blue and green zones, indicating that the main interaction was due to hydrogen bonding and van der Waals forces; these observations agreed well with the thermodynamic results of fluorescence analysis. It was also found that myricetin was completely encompassed by the isosurface at an isovalue of 0.001, while dihydromyricetin was encircled except for C5 and C6. The absence of the interaction at C5 and C6 was consistent with the molecular docking results. When the isosurface was set at 0.01 (Figure 4B1,B2), the interaction zone of myricetin was still significantly larger than that of dihydromyricetin. It could be concluded that the binding capacity of myricetin with α-glucosidase is superior to that of dihydromyricetin. As a result, its α-glucosidase inhibitory activity is higher than that of dihydromyricetin.

## 4. Conclusions

In this study, the α-glucosidase inhibitory activities of 15 common flavonoids were compared. The inhibitory performance of the flavonoid aglycones was significantly superior to that of the flavonoid glycosides. A positive relationship between the number of phenolic hydroxyl groups in the B ring of flavonoids and the activity was also determined. Myricetin and dihydromyricetin had similar structures, but the inhibitory activity of myricetin was significantly higher than that of dihydromyricetin. In the fluorescence analysis, myricetin also showed a higher binding capacity with α-glucosidase. To clarify the underlying mechanism, a molecular simulation of myricetin and dihydromyricetin was carried out. The molecular docking results suggested that they had different orientations in the active center of α-glucosidase. Myricetin exhibited stronger hydrogen bonding and van der Waals force interactions according to the IGM analysis, which was in agreement with the thermodynamic parameters. The results obtained in this study should prompt the application of flavonoids as hypoglycemic nutraceuticals. The proposed method of combining IGM with molecular docking analysis provides a convenient way to investigate the interaction between polyphenols and proteins.

## Figures and Tables

**Table 1:** The chemical structures and IC_50_ values (μg/mL) of 15 flavonoids.

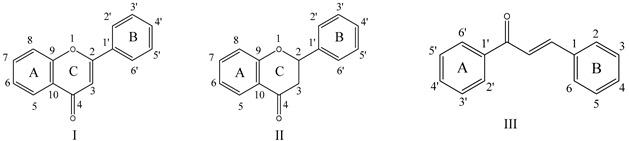												
**No.**	**Type**	**Name**	**3**	**4**	**5**	**6**	**7**	**2′**	**3′**	**4′**	**5′**	**IC_50_**
1	I	myricetin	OH		OH		OH		OH	OH	OH	2.09
2	I	fisetin	OH				OH		OH	OH		5.50
3	I	quercetin	OH		OH		OH		OH	OH		6.10
4	I	baicalein			OH	OH	OH					6.75
5	I	luteolin			OH		OH		OH	OH		27.22
6	III	isoliquiritigenin		OH				OH		OH		36.29
7	I	chrysin			OH		OH					70.07
8	II	dihydromyricetin	OH		OH		OH		OH	OH	OH	85.95
9	III	4-hydroxychalcone		OH								123.04
10	III	4′-hydroxychalcone								OH		199.10
11	II	hesperetin			OH		OH		OH	OCH3		286.60
12	II	naringin			OH		O-Rut			OH		>500
13	II	hesperidin			OH		O-Rut		OH	OCH3		>500
14	I	baicalin			OH	OH	O-Glu					>500
15	I	rutin	O-Rut		OH		OH		OH	OH		>500

(Rut, rutinoside; Glu, glucuronide).

**Table 2:** The quenching constants, binding constants, and thermodynamic parameters between two flavonoids and α-glucosidase at 298 and 303 K.

Flavonoids	T (K)	10^12^ K_q_ (L·mol^−1^·s^−1^)	10^4^ K_a_ (L·mol^−1^)	*n*	ΔG (KJ·mol^−1^)	ΔH (KJ·mol^−1^)	ΔS (J·mol^−1^·K^−1^)
Myricetin	298	9.6733	7.7786	0.9843	−27.91	−156.15	−430.33
	303	5.9984	2.7428	0.9386	−25.76		
Dihydromyricetin	298	3.1750	5.7637	1.0572	−27.16	−115.61	−296.81
	303	3.4092	2.6546	0.9764	−25.68		
